# The Use of Nonhuman Primate Models for Advancing HIV PrEP

**DOI:** 10.3390/v17091192

**Published:** 2025-08-30

**Authors:** Elena Bekerman, Christian Callebaut

**Affiliations:** Gilead Sciences Inc., Foster City, CA 94404, USA

**Keywords:** HIV, SIV, prevention, PrEP, nonhuman primate models

## Abstract

The global fight against HIV/AIDS has been significantly bolstered by the development and implementation of pre-exposure prophylaxis (PrEP), yet innovation in PrEP interventions, improved adherence and greater access are still needed to maximize its benefit. Nonhuman primate (NHP) infection with simian immunodeficiency virus (SIV) has served as an instrumental animal model in advancing HIV PrEP research. This review comprehensively examines the utility of NHP models in evaluating the efficacy, pharmacokinetics, and safety of diverse PrEP strategies, including oral, injectable, implantable, and topical formulations. It discusses the development of diverse challenge models that simulate human transmission routes and the advantages of NHPs in enabling controlled and mechanistically informative studies. It also highlights the successful translation of pivotal NHP studies evaluating tenofovir-based regimens as well the long-acting agents, cabotegravir and lenacapavir, into the clinical settings, emphasizing the consistently high predictive power of the NHP models for the HIV PrEP clinical efficacy. Finally, it underscores the importance of species-specific pharmacologic considerations and the value of NHP data in informing clinical trial design. As the global community strives to end the HIV epidemic as a public health threat in the absence of an efficacious prophylactic vaccine, NHP models make a critical contribution in the development of next-generation HIV prevention tools.

## 1. Introduction

HIV remains a significant public health challenge, with approximately 39 million people living with HIV (PLWH) worldwide and an estimated 1.3 million new infections occurring in 2023 [1]. Global fight against HIV/AIDS has made significant strides since the start of the epidemic with steep reductions in new infections where treatment options are available, and a record number of PLWH now achieving suppression on antiretroviral therapy. Yet, current success is not uniform across geographical regions and is still significantly short of the ultimate goal to end AIDS as a public health threat by 2030 [2]. Ensuring access to the available treatment and prevention options along with continued investment into treatment, prevention and cure research will be necessary to sustain the gains and ultimately reach that goal.

Pre-exposure prophylaxis (PrEP) is a powerful tool in the fight against HIV, though challenged by the limitations to access, social stigma, adherence and/or persistence. While development of PrEP has resulted in real-world decline in new cases in regions with high adoption rates [3], continued technological innovation of HIV PrEP can further accelerate global prevention by offering diverse choices that best suit individual needs and preferences. This review summarizes the features and use of non-human primate models for the investigation of new PrEP agents and how resulting data have translated clinically to date.

## 2. Macaque Models and Viral Strains

### 2.1. SIV vs. HIV

Simian models for HIV/AIDS have been instrumental in our understanding of the biology of HIV-1 infection and informing drug development, in particular for HIV PrEP and HIV cure efforts. HIV-1 can infect but not replicate in primate cells, owing to lack of adaptation to the primate restriction factors such as TRIM5alpha, APOBEC3, SAMHD1 and interferon-induced transmembrane (IFITM) proteins [4,5,6,7]. Simian immunodeficiency viruses (SIVs) do, however, cause chronic persistent infections of primate species that can effectively model human HIV-1 infection.

### 2.2. SIV Origins and Pathogenesis

SIVs, typically designated with a species-specific suffix to denote their non-human primate host origin (e.g., mnd, cpz, mac), are a family of lentiviruses that co-evolved in sub-Saharan African primates such as mandrills, chimpanzees, and others and gave rise to HIV-1 via an inferred zoonotic transfer [8]. Though largely non-pathogenic in their native hosts due to thousands of years of co-evolution, infection of Asian-origin macaques such as the rhesus macaque (*Macaca mulatta*), the pig-tailed macaque (*Macaca nemestrina*) and the cynomolgus macaque (*Macaca fascicularis*) with certain strains of SIV recapitulates the hallmarks of human AIDS. These include high levels of viral replication, chronic immune activation and inflammation, progressive CD4+ T cell depletion and opportunistic infections, albeit with more rapid progression in the macaque model [9].

### 2.3. Primate Model Species and SIV Variants

In addition to the similar pathogenicity and close phylogenetic relationship between SIV and HIV-1, macaques have anatomy, reproductive physiology, and immune systems similar to humans, making them the preferred species for human AIDS research. Rhesus macaques of Indian origin and pigtail macaques are the best characterized and most widely used species to evaluate viral transmission and prevention using either SIV or chimeric simian HIV (SHIV). SIV_mac251_, a genetically diverse viral swarm first isolated from rhesus macaques at the New England Primate Research Center in 1985, is a model virus with the broadest research usage [10]. A related SIV_mac239_ is a well characterized molecular clone derived from SIV_mac251_, which can be genetically manipulated and results in less variable infection parameters compared to the swarm [11]. Similarly to HIV-1, both SIV_mac239_ and SIV_mac251_ are CCR5-tropic and primarily target activated CD4+ T cells and macrophages during an acute infection. Chimeric SHIV viruses more closely resemble HIV-1, enabling more accurate modeling of the potency of clinical drug candidates in some cases, while still supporting robust viral replication in animal tissues. SHIVs are typically generated by recombination of select HIV-1 encoded genes into the backbone of an SIV vector and serial passage in animals to improve their replication capacity and pathogenicity. SHIV_SF162P3_, a pathogenic strain of SIV_mac239_ expressing an R5-tropic HIV-1 Env, resembles the dominantly transmitted HIV-1 and is also commonly used to evaluate transmission inhibitors [12]. To address the limitations of the SIV model for HIV-1 infection—such as differences in immune responses or drug sensitivity that are not adequately captured by SHIV chimeras—researchers have more recently developed minimally modified HIV-1 variants (mmHIV-1) [13]. Substitution of SIV *vif* and a macaque-adapted HIV-1 *env* into an HIV-1 clone, followed by serial passage in macaques, enabled selection of adaptive mutations that enhanced replication and pathogenesis, and resulted in mmHIV-1 strains like stHIV-A19. This strain has recently been used to probe prophylactic efficacy of a novel HIV-1 capsid inhibitor in vivo [14].

### 2.4. Distinct Routes of Transmission Models

Mucosal challenge animal models simulate the predominant routes of HIV transmission in humans, which occur through mucosal surfaces during sexual contact. These models encompass the natural mucosal routes of HIV transmission in humans, such as vaginal, rectal, penile, and oral exposures [15]. Rectal and vaginal models are the most commonly used models to study heterosexual and homosexual transmission routes as represented by the numerous examples discussed in this review. Viral challenges are typically accomplished via atraumatic inoculation of ~1 mL of virus-containing media into the rectal or vaginal vault via a slip tip syringe or a sterile gastric feeding tube. Chosen viral inoculums can be reported as multiples of animal infectious dose 50%, AID_50_ (e.g., 0.5 AID_50_), or tissue culture infectious dose 50%, TCID_50_ (e.g., 100 TCID_50_), or simply as a fold dilution of the viral preparation (e.g., 1:10).

Compared to rectal transmission, vaginal transmission rates are typically lower or more variable due to menstrual cycles and vaginal mucosal structure. Vaginal mucosa, which is composed of multiple layers of epithelium, provides a stricter physical barrier to infection, in contrast to a single layer of columnar epithelium in the rectum. Vaginal challenge studies have historically been less frequent due to both anatomical complexities and the limitations in the availability of female macaques in order to sustain breeding colonies across primate centers, but have increased in recent years [16]. To augment or normalize vaginal transmission rates researchers typically use higher viral titers to challenge the animals and often employ pre-treatment with an injectable hormonal contraceptive depot-medroxyprogesterone acetate (DMPA). Females treated with DMPA display dose-dependent temporal vaginal epithelial thinning with loss of the non-nucleated layer and an increase in susceptibility to infection [17,18,19]. Penile models were recently developed to study male-specific HIV acquisition and prevention strategies [20]. Two exposure routes tested atraumatic virus deposition at the inner foreskin, glans and urethral os or the distal urethra. Both routes resulted in dose-dependent rates of virus acquisition, while concurrent challenges via both routes produced 100% infection rates [20].

The intravenous (IV) route is the most reliable and consistent method to establish systemic infections. Typically, IV inoculation requires significantly lower viral doses to seed infection compared to mucosal routes. This model is used to mimic scenarios such as needle-sharing among intravenous drug users and blood transfusion-related HIV transmission. Additionally, pregnant macaques infected intravenously with SHIV demonstrated perinatal transmission to infants, highlighting the utility of IV models in studying maternal-fetal transmission [21]. Vertical transmission from an infected dam may occur in utero (typically very low rate of transmission), intrapartum or post-birth via breastfeeding [22]. Transmission via breastfeeding may also be modeled via direct oral inoculation of naïve infants with the virus.

### 2.5. Advantages of NHP Models for PrEP

The use of the simian models to understand pre- and post-exposure prophylaxis has numerous advantages. In PrEP trials, the background HIV incidence (the rate of new HIV infections in the study population without intervention) typically ranges from 2 to 10 per 100 person-years, depending on the study population’s risk factors [23,24,25]. Hence, to estimate the efficacy and reach their primary endpoints, typical PrEP trials enroll thousands of participants and span years until completion. Preclinical study designs, though aiming to maintain physiological relevance, typically utilize greater infectious dose viral inoculums and/or frequent repeat exposures yielding orders of magnitude greater infection incidences. Hence, preclinical studies can assess the infection risk reduction potential in a fraction of the time, with much smaller sample sizes than clinical trials. The timing of virus exposure relative to the drug administration and, thus, the drug exposure level can directly impact the extent of protection. Animal studies can help define the protective exposure threshold for a given drug by deliberately measuring the susceptibility to viral infection at or past the target C_trough_ drug concentrations. Susceptibility of viral strains or genetic variants to the antiretroviral agent can also influence the rate of infection and/or prophylaxis. All of these variables can be precisely controlled in a preclinical NHP study through the deliberate selection of timing, dose, and route of inoculation, as well as the viral strain or variant—factors that cannot be ethically manipulated in human trials. Moreover, invasive and frequent sampling, not easily feasible clinically, can offer insights into the viral and drug exposure kinetics and any relevant tissue-specific differences. Lastly, important ethical considerations that challenge the design or execution of modern HIV PrEP trials, such as inclusion of placebo controls, can be circumvented in preclinical research studies. And, as discussed below, nonhuman primate models have demonstrated a strong track record of translation to humans and continue to drive innovation in the PrEP space.

## 3. Earliest Utilization of NHPs for PrEP Research

Preclinical studies for PrEP and PEP in nonhuman primates date back to the early 1990s with azidothymidine (AZT or zidovudine/ZDV), a nucleoside reverse transcriptase inhibitor (NRTI) and the first approved antiretroviral for HIV treatment, evaluated in infant macaques [26] (Table 1). Van Rompay and colleagues demonstrated that AZT provided protection against infection when dosed every 8 h beginning 2 h prior to an intravenous inoculation with SIV in infant macaques. In contrast, a 6–10-week course of AZT exerted no detectable effect on the disease in established infection among animals that began treatment 6 weeks post-exposure. This study demonstrated the prophylactic potential of AZT in infants as well as highlighted the utility of the rhesus macaque model for antiretroviral drug testing. In 1994, data from the landmark PACTG076 clinical trial, which evaluated the reduction in maternal-infant HIV transmission risk with AZT, were published [27]. This double-blind placebo-controlled trial documented a dramatic 67.5% relative reduction in the risk of HIV transmission in HIV-affected pregnancies, thus corroborating the NHP data. These findings led to the global adoption of routine HIV testing for pregnant women, the implementation of the zidovudine regimen as the standard of care, and further research into simpler and more cost-effective regimens suitable for resource-limited settings [28]. While AZT is no longer the first-line treatment for HIV due to the availability of other safer, more effective, and convenient options, it remains an important tool in specific scenarios for preventing vertical transmission, particularly in resource-limited settings or as part of combination regimens.

## 4. TFV-Based PrEP

At the time of their development, tenofovir-based acyclic nucleotides, which require fewer phosphorylation steps to become active, represented the next generation of NRTIs developed against HIV. These drugs were also characterized by reduced susceptibility to resistance, and improved efficacy and safety profiles compared to zidovudine (AZT). An early preclinical study in juvenile macaques demonstrated that a 4-week course of daily PMPA, commonly referred to as tenofovir (TFV), prevented establishment of SIV infection in 100% of animals when initiated up to 24 h after an intravenous virus inoculation [29]. Importantly, the PMPA regimen was safer and more efficacious as compared to AZT in juvenile or adult macaques. Follow-on work revealed that extending the time to initiation of treatment from 24 to 48 or 72 h post-inoculation or decreasing the duration of treatment reduced the effectiveness of infection prevention, illustrating the correlation between the level of protection and drug exposure [30].

Tenofovir disoproxil fumarate (TDF), a prodrug of the parent TFV, was subsequently developed to improve permeability and absorption to allow for systemic delivery via oral administration. In 2001 TDF became the first tenofovir-based drug approved by the FDA for the treatment of HIV as a once daily pill. TDF is well tolerated and has a low rate of associated adverse events after long-term administration, which made it an attractive candidate to explore for the prevention of sexual HIV transmission. To that end, in the early 2000s researchers developed new mucosal models of transmission with repeat low dose (i.e., virus at the level comparable to HIV-1 present in human semen during an acute infection) SIV/SHIV exposures in adult macaques [51,52]. Compared to the previously explored high-dose intravenous or oral challenges designed to simulate vertical HIV transmission, the new models were more physiologically representative of sexual transmission to enable assessment of vaccines and microbicidal prophylaxis strategies. The first application of the low dose repeat exposure model evaluated the efficacy of daily or weekly oral TDF against rectal SHIV infection [31]. The results of this study suggested that TDF may reduce the overall rate of rectal transmission. However, the incomplete protection combined with the small study size and lack of statistical significance limited the interpretation and possible translation of this approach clinically. To build on this observation, another NHP study was undertaken to evaluate PrEP efficacy of regimens containing two ARVs, TFV or TDF and another NRTI emtricitabine (FTC) administered either orally or subcutaneously [33]. Though both drugs target the reverse transcriptase (RT) of HIV, their distinct and complementary resistance profiles and synergistic antiviral activity make them a highly effective antiretroviral combination. FTC monotherapy led to a 3.8-fold lower risk of infection compared to placebo, while dual therapy further reduced the risk to 7.8-fold with FTC/TDF or was completely protective in the case of FTC with high dose TFV. Together, these encouraging mucosal transmission NHP data, prior clinical validation of ARVs for PrEP with AZT in maternal-infant HIV prevention and a favorable safety profile established in the treatment of HIV justified the evaluation of TDF alone or in combination with FTC for sexual HIV prevention in adults.

Multiple randomized, double-blinded, placebo-controlled clinical trials of oral PrEP, prescribed for once-daily use, were undertaken to evaluate the safety and efficacy of TDF and/or TDF/FTC for HIV prevention [53]. The iPrEx trial conducted across multiple geographical regions between 2007 and 2011 demonstrated the efficacy of TDF/FTC among men who have sex with men (MSM), reducing HIV incidence by 44% [35]. Subgroup analyses revealed that detectable drug levels were strongly associated with substantially lower HIV risk. The Partners PrEP study similarly investigated the effectiveness of daily oral TDF and TDF/FTC vs. placebo for HIV prevention in HIV-serodiscordant couples in Kenya and Uganda between 2008 and 2010 [32]. This study reported a 67% reduction in the incidence of HIV-1 with TDF and 75% with TDF–FTC, and an association between protection and detection of tenofovir in participants’ blood samples. The TDF2 study conducted among heterosexual men and women in Botswana (2007–2010) with daily TDF/FTC reported 62% efficacy versus placebo and an increase to 78% among participants actively receiving the study drug [36].

These clinical trials collectively provided the evidence base for the FDA approval in July of 2012 and subsequent inclusion of Truvada (a fixed dose combination of FTC/TDF) in national and international guidelines for HIV prevention. Additional studies, including the FEM-PrEP and VOICE trials, which enrolled high-risk HIV-uninfected women across Africa between 2009 and 2012, discontinued early due to futility attributed to very low drug adherence (~30%) [37,38]. But despite their lack of efficacy, these studies provided valuable insights into adherence barriers and the importance of tailoring HIV prevention strategies to the social and cultural contexts of target populations.

## 5. TAF/FTC in NHP and DISCOVER

Similarly to TFV and TDF, nonhuman primate studies laid the foundation for the preclinical evaluation of the next generation prodrug of TFV, tenofovir alafenamide (TAF), for HIV prevention. TAF achieves target levels of the active metabolite (TFV diphosphate) at significantly lower TFV systemic exposure than TDF, which translates to improved safety parameters. Strong clinical efficacy and safety data led to the approval of TAF-based combination regimens for the treatment of HIV beginning in 2015 and made it an attractive candidate to evaluate for PrEP [54]. As such, Massud et al. demonstrated preclinically that short event-driven FTC/TAF regimens were protective similarly to FTC/TDF in both the rectal and vaginal challenge macaque models [41,45]. Specifically, macaques exposed rectally to SHIV on a weekly basis for up to 19 weeks remained fully protected with FTC/TAF regimen, while all the control animals became infected [45]. In the vaginal challenge model FTC/TAF provided 91% protection and delayed infection compared to controls in macaques exposed vaginally to SHIV once a week for up to 15 weeks [41]. Together, these studies supported clinical investigation of TAF-based regimens for PrEP use.

An active-controlled DISCOVER study among MSM and transgender women initiated in 2016, confirmed that daily prescribed TAF/FTC had non-inferior efficacy to TDF/FTC for HIV prevention and was ultimately approved by the FDA for PrEP in MSM and transgender women in 2019 [40]. Although, DISCOVER study did not explicitly evaluate efficacy in cisgender women, F/TAF was subsequently assessed in this population as part of the PURPOSE 1 study [34]. HIV incidence with F/TAF was not significantly lower than the background HIV incidence, and no meaningful difference in HIV incidence was observed between F/TAF and F/TDF, presumably due to the overall low drug adherence. However, the chance of acquiring HIV was 89% lower when adherence to F/TAF reached two pills per week or more [55].

## 6. Non-Daily PrEP

In addition to the daily schedules, some of the early preclinical NHP studies also explored more flexible dosing options with FTC/TFV in the context of weekly SHIV exposures. An intermittent schedule in macaques was fully protective with dosing 2 h before plus 24 h after each weekly virus challenge [33]. Follow on NHP work with oral FTC/TDF confirmed that simple regimens with one dose within 2 h of challenge plus one additional dose within 7 days pre-challenge or 26 h post challenge were similarly protective to the daily administration against both rectal and vaginal transmission [42,43,44]. Delay of start of dosing to 24 h post SHIV exposure resulted in loss of protection, underscoring the importance of blocking initial viral replication in the mucosa.

Using the NHP model, we and others have also explored the potential of augmenting chemoprophylactic activity of short-course NRTI regimens with an addition of integrase strand transfer inhibitors (INSTIs). Unlike NRTIs, which block the RT, INSTIs interfere with the viral DNA integration. Published models estimate strand transfer and integration of the HIV proviral DNA into the human genome to occur about 5 hours following the completion of reverse transcription which, depending on the experimental conditions, happens over the course of 6 to 48 hours post entry. Thus, the addition of INSTIs to the RT inhibitors has the potential to broaden the window of opportunity for a successful prophylactic intervention administered postexposure. Consistent with this hypothesis, Massud et al. highlighted the added value of a boosted INSTI, elvitegravir (EVG), to 1- or 2-dose F/TAF regimens by demonstrating a high degree of protection with initiation as late as 24 h post SHIV rectal challenge [50]. Specifically, they reported that while a single dose of FTC/TAF + boosted EVG was not protective as PEP, a two-dose regimen at 24- and 48 h post-exposure yielded a calculated efficacy of 77%. We similarly showed improved protection with the addition of an unboosted INSTI, bictegravir (BIC), to 2-dose F/TAF PrEP and PEP schedules using the rectal challenge macaque models [49]. Specifically, while FTC/TAF was significantly protective as PrEP, it lost efficacy as PEP. However, FTC/TAF plus BIC offered complete protection as PrEP and greater than 80% per-exposure risk reduction with treatment initiation up to 24 h postexposure.

Collectively, these studies demonstrated that drug loading closest to the time of virus exposure yields the highest protection, but the protective window may be expanded by the addition of an INSTI to the NRTI regimen. This evidence also highlighted the promise of non-daily alternatives to the daily oral PrEP with potential benefits such as better alignment to sexual practices, reduced pill fatigue, cost-effectiveness, and reduced drug toxicity.

Event-driven prophylaxis efficacy was ultimately validated clinically among men who have sex with men (MSM) through the IPERGAY trial [24]. The trial design was largely informed by the 2-2 regimen (i.e., double dose of Truvada at 40 mg/kg FTC and 44 mg/kg TDF given before and after virus exposure) found to be highly protective in the macaque model [42]. IPERGAY results demonstrated an 86% infection risk reduction with Truvada (TDF/FTC) prescribed at a 2-1-1 schedule (i.e., two pills taken 2–24 h before sex and one pill each taken 24 and 48 h after the first dose). IPERGAY trial participants reported high adherence to the dosing schedule, with a median of 16 pills per month. However, additional trials exploring the safety, adherence and acceptability of intermittent Truvada found adherence to time-driven or event-driven dosing to actually be lower compared to daily dosing [39,46,47,48]. Thus, while the flexibility of non-daily oral PrEP like Truvada use may be appropriate for certain populations with infrequent or predictable sexual activity, daily PrEP is superior for improved coverage of sex acts. Clinical trials and real-world studies have yet to confirm the protective efficacy of non-daily FTC/TAF or INSTI-containing oral regimens for PrEP in humans.

## 7. Long-Acting PrEP

### 7.1. INSTIs

Long acting injectables represent the newest frontier both for the treatment and prevention of HIV. The advantages of this modality, as precedented by the successful implementation of long-acting parenteral injections in contraception and antipsychotic treatment, may include improved adherence, convenience, reduced stigma and expanded access. As with the oral PrEP options, the development of long-acting PrEP is also rooted in the efficacy demonstration using the nonhuman primate models (Table 2).

Cabotegravir (CAB or GSK744), an analog of dolutegravir (DTG), is an INSTI with physiochemical properties that permit its formulation as an injectable nanosuspension dosed every 1 to 2 months. As safety and efficacy of both oral and parenteral CAB was being validated clinically for the treatment of HIV, which ultimately resulted in its approval in combination with rilpivirine to treat HIV, preclinical models began to examine CAB’s efficacy for PrEP. Repeat low-dose challenge models designed to more closely mimic HIV-1 transmission in humans, demonstrated protection from intrarectal or intravaginal SHIV challenges in macaques dosed intramuscularly with CAB on a once-monthly schedule [56,59]. Notably, the protection was complete at drug exposures achieved clinically with quarterly injections and declined in correlation with the plasma drug decline during the washout period. The correlate of complete protection in this study was plasma CAB concentrations >3x paIC_90_. A follow-up long-acting CAB repeat high-dose vaginal challenge model that also utilized DMPA pre-treatment retained significant protection, albeit not complete [87]. Specifically, monthly CAB injections protected 6 of 8 female macaques against three high-dose SHIV challenges, whereas all control animals became infected after the first challenge. Long-acting CAB was also tested in parallel with oral FTC/TDF against repeated penile SHIV transmission and both regimens were found to be highly effective at 94% protection [57].

Study HPTN 083 ultimately demonstrated that CAB administered every 8 weeks was superior to daily oral TDF-FTC in preventing HIV infection among cisgender men and transgender women who have sex with men. HIV incidence was reduced by 66% [61]. Inadequate TDF-FTC adherence among some participants appeared to drive the overall finding of higher HIV incidence than in the CAB group. Study HPTN 084 also showed CAB’s superiority over TDF-FTC among cisgender women in sub-Saharan Africa, with an 89% reduction in HIV incidence [60]. These data supported the approval of CAB extended-release injectable suspension for HIV PrEP in adults and adolescents in 2021.

### 7.2. CAIs

Lenacapavir (LEN) is a first-in-class capsid inhibitor with picomolar potency against HIV-1 and physicochemical properties that enable parenteral dosing once every 6 months with a potential for extended dosing interval of once yearly [88,89]. The safety and potency of LEN resulted in its approval for the treatment of heavily treatment-experienced adults with multidrug-resistant HIV-1 infection in combination with other antiretroviral agents. The first evidence of capsid inhibitor monotherapy prophylaxis was demonstrated in the rectal challenge SHIV model using an analog of LEN, GS-CA1 [62]. That study demonstrated that a single subcutaneous administration of GS-CA1 provided per-exposure infection risk reduction of up to 97% against 15 weekly challenges. Notably, GS-CA1 levels greater than twice the rhesus plasma paEC_95_ concentration were estimated to confer 100% protection in this model. Follow-on work extended these findings to the repeat vaginal challenge model, demonstrating similar efficacy [90]. However, the lag time between infection and detection—particularly in the presence of an antiviral—limits the accurate determination drug levels at the time of seeding of an infection, and thus constrains the estimation of protective drug concentrations using the repeat challenge model. To overcome this limitation, a subsequent study using LEN focused on examining the efficacy at clinically relevant drug exposures using a high-dose single rectal challenge model [63]. Complete protection and superiority to the untreated group was observed among animals whose LEN plasma exposure exceeded its model-adjusted clinical efficacy target at the time of exposure. Additionally, an intravenous challenge model with simian-tropic HIV (stHIV-A19) similarly demonstrated complete protection with a SC LEN injection, comparable to the daily treatment with TDF/FTC/DTG regimen [14]. Together, these preclinical studies supported continued clinical evaluation of LEN for PrEP, including in people who inject drugs.

The PURPOSE-1 study conducted among adolescent girls and young women in South Africa and Uganda showed 100% efficacy of twice-yearly LEN injections in preventing HIV and superiority compared to HIV background incidence and daily oral FTC/TDF [34]. In the PURPOSE-2 study, which included cisgender men, transgender individuals, and gender-nonbinary persons across multiple geographical regions, new HIV infection incidence in the LEN-treated group was 96% and 89% lower than the HIV background incidence and the incidence with FTC/TDF, respectively [64]. Collectively these data supported the FDA’s approval in 2025 of twice-yearly lenacapavir for PrEP in the United States, marking a potentially transformative moment in HIV prevention by offering very real opportunity to help end the HIV epidemic. Ongoing PURPOSE 3, 4, and 5 trials will complement the pivotal trials with additional populations including people who inject drugs [91]. A series of additional global regulatory applications have been filed or planned to enable broader access, including in settings with the highest HIV incidence.

### 7.3. Broadly Neutralizing Antibodies

Broadly neutralizing antibodies (bNAbs) are antibodies targeting conserved regions of the HIV envelope protein and capable of neutralizing a wide range of HIV strains and mediating infected cell killing. First-generation bNAbs developed in the 1990s, showed modest potency and breadth, with rapid emergence of resistant viral variants [92]. However, advances in antibody isolation techniques have led to a new generation of bNAbs with significantly improved potency and broader coverage, offering greater promise for both HIV-1 prevention and therapy.

The first-generation bNAb combinations demonstrated the initial preclinical proof-of-concept for HIV prophylaxis. These included 2F5 and 2G12, used in combination with the polyclonal human immunoglobulin derived from HIV-1 infected donors, conferring protection in both the intravenous and mucosal challenge models in adult macaques [66,68]. Examining vertical transmission, the same 2 bNAbs in combination with an antibody F105 also demonstrated protection against IV challenge in pregnant female macaques and against oral mucosal challenge in neonates [67]. Subsequent studies were able to achieve effective prophylaxis with just single bNAbs (e.g., b12, 2F5, 4E10 or 2G12), but required particularly high doses difficult to achieve or sustain clinically [73].

The next generation of highly potent bNAbs such as PGT121 demonstrated protection against mucosal challenge with SHIV at doses resulting in just single-digit µg/mL serum exposures [69]. Subsequent studies confirmed these findings and further characterized the mechanism of antibody-mediated protection. Despite complete protection, low levels of viral RNA and DNA were detected in distal tissues for seven days following challenge and cells from these tissues resulted in productive infection upon adoptive transfer to naïve hosts. These data suggest that the bNAb mechanism of protection may involve the clearance of infectious virus in distal tissues, in addition to preventing the seeding of the infection, which is why bNAbs are being evaluated for both HIV-1 treatment and prevention [72]. Other new generation bNAbs (e.g., VRC01, 3BNC117, 10-1074) alone or in combination also effectively protected monkeys from repeated mucosal or IV virus exposures in a manner that correlated with their potencies and half-lives paving the way for clinical testing [65,70,71].

Two phase 2b AMP trials, HVTN 703/HPTN 081 and HVTN 704/HPTN 085, recently evaluated the efficacy of bi-monthly intravenous infusions of VRC01, a CD4-binding site bNAb, for HIV-1 prevention in people [75]. VRC01 intervention did not reduce the overall rate of HIV-1 acquisition; however, post hoc analysis revealed that this bNAb could effectively prevent infection by VRC01-sensitive viruses. Together, these trials provided a proof-of-concept for bNAb-mediated prevention and helped define the appropriate bNAb target exposures relative to their in vitro potency [93]. However, they also underscored the need for antibody combinations to optimally cover globally circulating HIV-1 subtypes. Certain dual and triple combinations have already been derisked for safety and PK in phase 1 trials, laying the foundation for the design of future efficacy trials [74]. Additionally, with advances in protein engineering, efforts including Fc modifications or multi-specific formats are underway to develop bNAbs with further enhanced potency, breadth, and half-life.

### 7.4. NRTTIs

Islatravir (ISL), also known as MK-8591 or EFdA, is a first-in-class reverse transcriptase–translocation inhibitor (NRTTI), which due to its high potency and long intracellular half-life, enables infrequent oral administration at low doses. Preclinical studies evaluated weekly oral administration of ISL as protection from intrarectal challenges with SHIV [77]. This study identified a weekly dose of ISL as low as 0.43 mg/kg to be fully protective, while lower doses of 0.1 and 0.025 mg/kg were partially or non-protective, respectively. These results helped estimate a benchmark for prophylactic EC_90_ for ISL as 24 fmols of islatravir-triphosphate per million PBMCs, which was achieved with a low 0.1 mg/kg dose. Additionally, ISL’s efficacy as PEP option has been explored using an intravenous SIV challenge macaque model [76]. Two weekly doses of ISL given 24 h after intravenous challenge completely prevented infection, although a single dose was insufficient for protection. Using a subdermal implant for sustained drug delivery Pons-Faudoa and colleagues achieved sustained preventative concentration of ISL in the blood and tissues of nonhuman primates for 20 months and 100% protection against vaginal or rectal challenge with SHIV [85].

These compelling preclinical prophylaxis data combined with positive clinical data for the HIV treatment prompted the launch of ISL PrEP trials in diverse populations examining both the monthly oral doses and once-yearly implant modalities [78,79]. However, in 2021, the FDA placed clinical holds on all ISL trials after lymphopenia was observed across several clinical studies. While the studies evaluating ISL in combination with other agents for the treatment of HIV-1 have resumed with dose adjustment, the development of ISL has been discontinued for PrEP.

A structurally novel NRTTI, MK-8527 with PK suitable for less-frequent-than-daily dosing and comparable antiviral activity to ISL has now been described [94]. It was recently tested as a weekly oral prophylaxis in a repeat rectal challenge macaque model [80]. No infections occurred in any of the animals dosed with MK-8527 (0.1–6 mg/kg), while 7 out of 8 or 5 out of 8 macaques in the undosed and the vehicle only dosed groups, respectively, became infected after 3–10 challenges. This translated to an 11- to 18-fold lower risk of infection with MK-8527 compared to the controls. Based on the PK efficacy threshold derived from the macaque study combined with phase 1 PK and safety data, 3 once-monthly oral dose levels of MK-8527 (3, 6 and 12 mg) were selected for evaluation in a phase 2 trial, with data disclosure still pending (NCT06045507). Furthermore, two Phase 3 trials—NCT07044297 and NCT07071623—have been announced to evaluate the safety and efficacy of once-monthly oral MK-8527, with enrollment projected to begin in August 2025.

### 7.5. TAF Implants

Efforts toward long-acting PrEP have also involved the preclinical development of biodegradable or non-biodegradable polymeric implants for sustained delivery of TAF. A subcutaneous nanofluidic implant achieved above clinically protective active metabolite drug levels and conferred 62.5% infection reduction against repeated low-dose rectal SHIV challenge in macaques with just slight tissue reactivity response [83]. A different efficacy study was conducted with subcutaneously administered polycaprolactone implants (0.7 mg/day TAF release rate) in a macaque model challenged vaginally twice weekly [84]. TAF implants completely prevented vaginal SHIV acquisition; however, local tissue inflammation and necrosis were observed near the implant sites. These findings were consistent with an additional report documenting adverse local inflammation and necrosis near the implant in macaques with even lower TAF release rates [82].

CAPRISA 018 Phase I clinical trial tested the acceptability of an annual 110 mg TAF silicone implant for HIV prevention in South African women [86]. While implant attributes, physical experiences and insertion/removal procedures were largely acceptable, local implant-site reactions significantly reduced tolerability, as predicted by the macaque model, resulting in higher-than-expected early removals. Taken together, the mechanisms of local toxicities associated with TAF-releasing implants must be better understood and improved before they can become a viable PrEP strategy.

## 8. Topical PrEP Options

Topical HIV prevention strategies, often referred to as microbicides, involve applying formulated antiviral agents directly to mucosal surfaces to prevent HIV transmission. They represent a highly desirable option among individuals who prefer an on-demand HIV prevention option that can be easily and discretely self-administered. Delivery of microbicide to the mucus membranes lining the surface of the vagina or rectum can be accomplished through materials such as vaginal rings, gels, films, inserts, suppositories, or foams. Because they deposit active ingredients directly at the site of HIV exposure, topical agents generally use small amounts of drug, avoid side-effects associated with systemic delivery, and may have reduced requirements for clinical monitoring. Although no such products are currently marketed outside of low- and middle-income countries, research is actively aimed at identifying safe, effective, affordable and desirables microbicides for HIV prevention (Table 3).

Early clinical studies involving detergents or polyanionic microbicide products such as Carraguard, PRO 2000, Cellulose Sulfate, and Nonoxynol-9 proved ineffective or even enhanced infection in certain cases [114]. The latter was later attributed to the interaction of polyanionic microbicides with semen-derived amyloid fibrils, which enhance HIV infectivity rather than inhibiting it [115]. Subsequent efforts pivoted to antiretroviral-based microbicides, which can offer a more targeted approach to HIV prevention.

Cranage et al. were first to evaluate rectal application of 1% TFV gel using a rectal SIV challenge macaque model [97]. They demonstrated that 4 out of 6 and 2 out of 3 macaques pre-treated with TFV gel 15 min and 2 h prior to the challenge, respectively, were protected, whereas 7 out of 8 control animals became infected. Furthermore, the analysis of plasma TFV concentration at the time of virus challenge revealed rapid absorption and a strong association between drug concentration and protective efficacy. Similar NHP study demonstrated that a vaginal application of gel with 1% TFV alone or in combination with 5% FTC 30 min prior to challenge fully protected macaques from a total of 20 SHIV exposures [99]. Earlier study using higher doses of TFV gel (10% weight per weight) administered intravaginally at four timepoints (24 h before, 0 h, 24 h after and 48 h after inoculation), was also fully effective in preventing SIV transmission after repeated intravaginal challenge [96,116]. The drugs were similarly rapidly absorbed and detected in the plasma 30 min after application. Because of the long intracellular half-life of TFV and high drug exposure in vaginal tissues, researchers hypothesized that a vaginal gel containing TFV may provide long lasting protection. To that end, Dobard et al. delayed vaginal SHIV challenges until 3 days after gel application and demonstrated protection in 4/6 macaques [95]. Moreover, this group concluded that TFV-DP concentrations above the in vitro IC_95_ in vaginal lymphocytes was a good predictor of high efficacy encouraging future evaluation of improved delivery methods of topical TFV.

In early clinical validation, a phase 2b proof-of-concept trial of pericoital intravaginal TFV 1% gel (CAPRISA-004) provided evidence that tenofovir use could safely reduce the risk of HIV acquisition by sexually active women in South Africa [100]. The dosing strategy required application of the gel both before and after coitus, resulting in HIV infection reduction by 39% overall, and by 54% in women with high adherence to the protocol. The 2010 AIDS conference presentation of CAPRISA-004 clinical data marked a pivotal moment in HIV prevention, providing compelling proof of concept for microbicides. Furthermore, the findings clinically validated the NHP model system and laid the groundwork for subsequent studies involving TVD and TDF. Unfortunately, low adherence significantly impacted efficacy in the subsequent phase 3 trials, VOICE and FACTS 001, which ultimately failed to demonstrate protection with either daily or pericoital (one dose before plus one dose after sex) use protocols [38,103].

As a more practical alternative to microbicide gels, vaginal or rectal inserts are a discreet, portable, and easy to self-administer option for HIV prophylaxis. Inserts can be used for both pre-exposure prophylaxis (PrEP) and post-exposure prophylaxis (PEP), providing a wider window of protection compared to gels. Rectal inserts with placebo formulation also scored high in acceptability and adherence in a recent randomized trial among young sexual and gender minorities [117]. CONRAD/EVMS (Arlington and Norfolk, VA, USA) have developed fast-dissolving inserts containing TAF and an INSTI, elvitegravir (EVG) for on-demand vaginal or rectal pericoital use. Preclinical studies demonstrated safety and pre- and postexposure protection of the TAF/EVG 20 mg/16 mg insert given vaginally or rectally within 4 h of viral challenge in NHP models [107,108]. Follow on nonhuman primate study reported that the insert application 8 h or 24 h after exposure maintains a high degree of efficacy preclinically [106]. Although, individual contributions of TAF or EVG were not determined, the authors speculated that rapidly achieved high levels of EVG in mucosal tissues prevented virus integration, while slower-rising but sustained high levels of TFV-DP maintained a continued inhibition of HIV replication. Phase 1 clinical study validated that rectal administration of TAF/EVG inserts achieves high rectal tissue concentrations of EVG and TFV-DP with low systemic drug exposure and demonstrable ex vivo inhibition of HIV infection for 72 h [109]. Future studies with longer dosing durations of weeks or months will be needed to establish the safety and acceptability with longer-term use.

Dapivirine (DPV) is another potent HIV-1 RT inhibitor which belongs to the class of NNRTIs. Its oral bioavailability and high lipophilicity make DPV a suitable candidate for topical delivery. Studies in rhesus macaques demonstrated that intravaginal administration of DPV gel resulted in high concentrations of the drug in vaginal and cervical tissues [98]. Drug-related material was observed in the keratinized and superficial cellular layers of the vaginal epithelium, and in some cases, extended into deeper layers. Though PrEP efficacy was not directly evaluated in the macaque model, the persistence of biologically significant concentrations of DPV in vaginal and cervical tissues for >24 h supported clinical evaluation of DPV as a microbicide.

A monthly silicone matrix vaginal ring containing 25 mg of DPV was ultimately assessed in two phase 3 clinical trials. MTN-020/ASPIRE and IPM 027/The Ring studies both demonstrated good tolerability and a 35% and 27% reduction in the risk of HIV acquisition, respectively [101,102]. Similarly to the trials with oral PrEP, higher efficacy was observed in subgroups with evidence of increased adherence. Results from the open-label extension studies of the trials showed increases in ring use and modeling data suggested even greater risk reduction—by over 50% across both studies—compared to the Phase 3 trials [104,105]. The European Medicines Agency (EMA) adopted a positive scientific opinion on the monthly DPV vaginal ring in 2020 and the World Health Organization (WHO) subsequently recommended it as an HIV prevention method for women at substantial risk of HIV infection. It is currently available in several African countries. The ring is also being investigated for a longer duration (3-month) version.

Maraviroc, a CCR5 antagonist HIV entry inhibitor approved for the treatment of HIV infection is another agent evaluated for topical protection against infection acquisition. Vaginal application of maraviroc gel provided dose-dependent protection against SHIV acquisition in macaques, with complete protection achieved at higher concentrations [111]. Rectal-specific maraviroc gel formulations demonstrated high efficacy (82%) in preventing rectal SHIV transmission in macaques over multiple challenges [110]. To date, only oral maraviroc has been evaluated clinically for HIV prevention with 2 Phase 2 studies in MSM and women demonstrating safety and tolerability over 48 weeks but no statistical power to assess the efficacy [112,113]. However, preclinical studies have demonstrated that oral maraviroc lacks prophylactic efficacy in macaques, despite high drug concentrations in rectal tissues, arguing against further pursuit of this approach for PrEP [118].

Another antiviral explored for topical delivery is an NNRTI UC781, whose high hydrophobicity and low solubility are not well suited for aqueous formulation. Gel formulations of UC781 were evaluated in macaque models for vaginally and rectally applied topical microbicides. Research found no systemic absorption after vaginal or rectal application of UC781 formulations, with minimal impact on tissue integrity and microflora [119]. A Phase 1 trial tested UC781 gel for rectal application and found good safety, no systemic absorption, and high acceptability. Ex vivo biopsy infections from that study demonstrated marked suppression of HIV infectibility. Similarly, a phase 1 trial in women using UC781 vaginal gels demonstrated safety, minimal systemic absorption, and high acceptability [120]. However, challenges in formulation development and stability have hindered its development since.

## 9. Considerations for Translational HIV Prophylaxis Research in NHPs

The NHP model has been instrumental in laying the groundwork for the application of ARVs for human HIV prevention. Yet, as with any preclinical model, a wide array of variables must be taken into account to infer its predictive value. The differences in drug pharmacokinetic parameters such as absorption, distribution, metabolism, and excretion (ADME) between species should be considered. Factors such as gastrointestinal pH, transporters, and first-pass metabolism can all influence drug absorption, the process by which a drug enters the bloodstream following oral administration. Factors such as tissue perfusion, plasma protein binding, and lipophilicity affect drug distribution within the body. The metabolism impacts the availability of active drug levels by generating active or inactive metabolites. Lastly, excretion describes the removal of a drug and its metabolites from the body, primarily via the kidneys or liver. All of these parameters can differ due to genetic or physiological species differences as well as disease states. For example, nonhuman primates often exhibit comparable absorption, but higher metabolic rates and clearance compared to humans [121]. The interplay between these parameters will determine the systemic and tissue specific drug concentrations, which in turn, will influence both the therapeutic efficacy and potential toxicity. As an example, we previously reported higher plasma protein binding (i.e., lower free active drug fraction) and shorter half-life for bictegravir in macaques in contrast to humans, which implies that higher drug doses may be required in this preclinical model to achieve comparable pharmacologic activity [49].

Antiviral activity is typically reported as an EC_50_ value, concentration in cell culture media that provides 50% maximal antiviral activity, which can be influenced by the viral strain, genetic resistance profile, cell line or culture conditions, analytical assay, etc. The EC_50_ can then be used to derive EC_90-95_ and further adjusted for species plasma protein binding to calculate the in vivo target trough concentrations (C_trough_) for antiretroviral drugs. These targets are typically represented as multiples of plasma protein binding adjusted EC_90-95_ (paEC_90-95_). For example, in the case of evaluating LEN’s potential for PrEP in macaques at clinically relevant exposures, we computed the macaque model–adjusted clinical minimum target exposure based on the potency difference between SHIV in primary rhesus cells and HIV in human cells adjusted for differences in rhesus and human plasma protein binding of LEN. While breakthrough SHIV infections occurred at low systemic drug exposures, LEN was fully protective above the model-adjusted clinical drug level targets [63].

Overall, the predictive value of NHP PrEP studies with SIV/SHIV can be maximized through careful dose conversion based on the multiple species- and virus-adjusted parameters.

## 10. Concluding Remarks

Despite decades of intensive research, HIV prophylactic vaccines have yet to demonstrate efficacy in phase III clinical trials. Concurrently, the scale-up of PrEP implementation in resource-limited settings continues to face significant barriers. To achieve the global target of ending AIDS as a public health threat by 2030, it is imperative to accelerate HIV prevention efforts. This necessitates a comprehensive approach that integrates behavioral and structural strategies tailored to the needs of specific populations with diverse and effective biomedical interventions enabled by widely implemented global drug access mechanisms.

As regulatory agencies increasingly advocate for the reduction or replacement of animal testing with alternative, human-relevant methods—such as AI-based computational approaches—these novel strategies still require translational validation. To date, non-human primates (NHPs) remain the most robust and relevant preclinical model for HIV prevention research. The majority of foundational human studies evaluating ARV efficacy for HIV prevention have been predicated on preclinical demonstrations of effectiveness in NHPs. These models provide a comprehensive framework for estimating HIV PrEP efficacy, determining target doses, and identifying potential adverse effects—capabilities not feasible with lower-order animals such as rodents. These models’ suitability for longitudinal studies and invasive procedures, including tissue sampling, often enhances our mechanistic understanding of successful interventions. Importantly, NHP PrEP models have shown a remarkable ability to predict the clinical success of the currently approved PrEP agents and, conversely, to preclinically screen out approaches unlikely to succeed, reinforcing their critical role in translational HIV prevention research. While ethical considerations and interspecies variability must be carefully accounted for, NHP models have been instrumental in advancing the field of HIV prevention and will likely continue to bridge the translational gap between basic research and clinical application of PrEP.

## Figures and Tables

**Table 1:** Summary of oral HIV PrEP Strategies Evaluated in NHP Models.

PrEP Strategy		Drug(s)	Preclinical NHP Challenge Model(s)	Preclinical References	Clinical Translation	Clinical References
Oral Daily	Single agent NRTI	AZT, TFV, TDF	IV/Rectal	[26,29,30,31]	PACTG076 trial	[27,32]
	NRTI dual combination	TFV/FTC, TDF/FTC, TAF/FTC	Rectal/Vaginal	[33]	iPrEx; Partners PrEP; TDF2; FEM-PrEP; VOICE; DISCOVER	[32,34,35,36,37,38,39,40]
Oral Intermittent	NRTI dual combination	TFV/FTC, TDF/FTC, TAF/FTC	Rectal/Vaginal	[33,41,42,43,44,45]	IPERGAY	[24,39,46,47,48]
	NRTI + INSTI	TAF/FTC + BIC/EVG	Rectal	[49,50]	Not yet validated clinically	

Abbreviations: AZT, Zidovudine; BIC, Bictegravir; EVG, Elvitegravir; FTC, Emtricitabine; INSTI, Integrase strand transfer inhibitor; IV, Intravenous; NRTI, Nucleoside reverse transcriptase inhibitor; PO, Oral; TAF, Tenofovir alafenamide; TDF, Tenofovir disoproxil fumarate; TFV, Tenofovir.

**Table 2:** Summary of long-acting HIV PrEP Strategies Evaluated in NHP Models.

PrEP Strategy	Drug(s)	Dosing Route	Preclinical NHP Challenge Model(s)	Preclinical References	Clinical Translation	Clinical References
INSTI	CAB	IM	Rectal/Vaginal/Penile/IV	[56,57,58,59]	HPTN 083/084	[60,61]
CAI	GS-CA1, LEN	SC	Rectal/Vaginal/IV	[14,62,63]	PURPOSE 1/2	[34,64]
bNAb	b12, 2F5, 4E10, 2G12, VRC01, PGT121, 3BNC117, 10-1074, others	IV	Rectal/Vaginal/IV	[65,66,67,68,69,70,71,72,73]	HVTN 703/HPTN 081; HVTN 704/HPTN 085	[74,75]
NRTTI	ISL	PO	Rectal/IV	[76,77]	IMPOWER 22; IMPOWER 24; development halted	[78,79]
NRTTI	MK-8527	PO	Rectal	[80]	development pending	
NRTI, NRTTI	TAF, ISL	Implant	Rectal/Vaginal/IV	[81,82,83,84,85]	CAPRISA-018	[86]

Abbreviations: bNAb, broadly neutralizing antibody; CAB, cabotegravir; CAI, capsid inhibitor; IM, intramuscular, INSTI, integrase strand transfer inhibitor; ISL, islatravir; IV, Intravenous; LEN, lenacapavir; NRTI, nucleoside reverse transcriptase inhibitor; NRTTI, nucleoside reverse transcriptase translocation inhibitor; PO, oral; SC, subcutaneous.

**Table 3:** Summary of topical HIV PrEP Strategies Evaluated in NHP Models.

PrEP Strategy	Drug(s)	Formulation	NHP Challenge Models	Preclinical References	Clinical Translation	Clinical References
N(N)RTI gels	TFV, DPV	Gel/Ring	Rectal/Vaginal	[95,96,97,98,99]	CAPRISA-004; VOICE; FACTS; MTN-020/ASPIRE; IPM 027/The Ring	[38,100,101,102,103,104,105]
NRTI + INSTI inserts	TAF/EVG	Insert	Rectal/Vaginal	[106,107,108]	Ph1	[109]
Entry inhibitors	Maraviroc	Gel/PO	Vaginal/Rectal	[110,111]	Ph2	[112,113]

Abbreviations: DPV, dapivirine; EVG, elvitegravir; N(N)RTI: nucleoside (or non-nucleoside) reverse transcriptase inhibitor; PO, oral; TAF, tenofovir alafenamide; TFV, tenofovir.
